# Supplementary material for: An RNA helicase coordinates with iron signal regulators to alleviate chilling stress in Arabidopsis
Source: Nat Commun. 2025 Apr 29;16:3988. doi: 10.1038/s41467-025-59334-9 (PMC12037725; doi:10.1038/s41467-025-59334-9)
Supplement: Supplementary file 1 — Supplementary Information [file 41467_2025_59334_MOESM1_ESM.pdf]

Supplementary information for

An RNA helicase coordinates with iron signal regulators to alleviate chilling stress in Arabidopsis

Yingying Xing[1], Yawen Li[2], Xinmeng Gui[3], Xianyu Zhang[1], Qian Hu[1], Qiqi Zhao[1], Yongli Qiao[3], Ning Xu[1,*], and Jun Liu[1,*]

[1]State Key Laboratory of Agricultural and Forestry Biosecurity, MOA Key Lab of Pest Monitoring and Green Management, College of Plant Protection, China Agricultural University, Beijing 100193, China

[2]State Key Laboratory of Plant Genomics, Institute of Microbiology, Chinese Academy of Sciences, Beijing 100101, China

[3]Shanghai Key Laboratory of Plant Molecular Sciences, College of Life Sciences, Shanghai Normal University, Shanghai 200234, China

*E-mail: Junliu@im.ac.cn; xn741852@163.com

Supplementary Fig.1

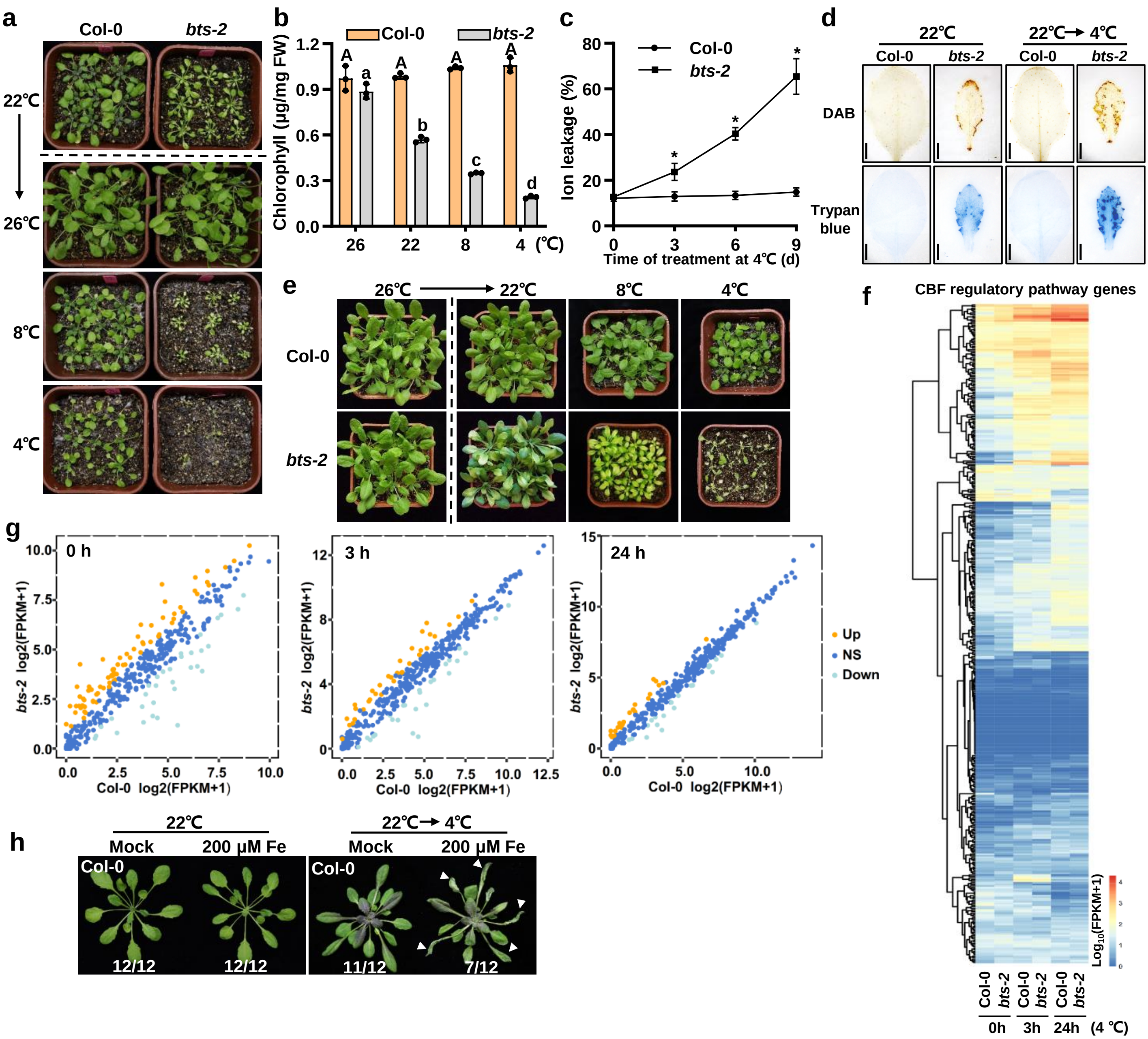

Supplementary Fig. 1 Chilling sensitivity of *bts-2* mutants.

(**a**) *bts-2* mutant is sensitive to low temperatures. The Col-0 and *bts-2* plants were grown in soil at 22℃ for 2 weeks and then transferred to 26, 22, 8 or 4℃ for another 1 week, respectively. The representative phenotypes were shown.

(**b**) Chlorophyll concentration of Col-0 and *bts-2* seedlings in (**a**). Six plants were used for per replicate. Data presented as means ± SD (n=3 biological replicates) by two-factor ANOVA with Tukey's HSD test ($P \leq 0.05$).

(**c**) Ion leakage assays for *bts-2* plants. Plants were grown at 22℃ for 2 weeks and then were treated at 4℃ for the indicated times. Ten individual plant leaves were used for per replicate at each time point. Data presented as means ± SD (n=3 biological replicates) by two-factor ANOVA with Tukey's HSD test (*$P \leq 0.05$).

(**d**) DAB and trypan blue staining of *bts-2* plants. Col-0 and *bts-2* plants were grown at 22℃ for 4 weeks and transferred to 4℃ for another 2 days, and then the leaves were stained with DAB and trypan blue, respectively. Bar = 1 mm. The experiment was repeated at least three times with six individual leaves each time.

(**e**) Pretreatment at 26℃ did not reduce the sensitivity of *bts-2* to low temperatures. Plants were grown at 26℃ for 2 weeks and then transferred to 26, 22, 8, or 4℃ for additional 2 weeks, respectively. The representative phenotypes were shown.

(**f**) The expression of CBF-regulated genes in *bts-2* mutants under chilling stress. Col-0 and *bts-2* plants were grown at 22℃ for 4 weeks and then subjected to 4℃ treatment for 3 h or 24 h. The plant leaves were sampled for transcriptome assays. There were no significant changes of the selected CBF-responsive genes between Col-0 and *bts-2* at the same time point (3 h or 24 h). Two biological replicates were performed for each sample at each time point.

(**g**) The CBF pathway-related gene expression was not affected in *bts-2* mutant under chilling condition. FPKM scatter plot of CBF-regulated genes under 4℃ treatment for 0 h, 3 h and 24 h in (**f**). The log2(FPKM+1) of Col-0 samples was set as the x-axis, and that of *bts-2* samples was set as the y-axis. NS, not significant.

(**h**) Oversupply of iron undermines plant chilling tolerance. Plants were grown in soil (Mock) or supplemented with 200 μM Fe-EDTA at 22℃ for 4 weeks, and then the plants were treated at 4℃ for 14 days. Left panel showing the representative plants before treatments. Numbers indicate the representative phenotype.

Source data are provided as a Source Data file.

Supplementary Fig.2

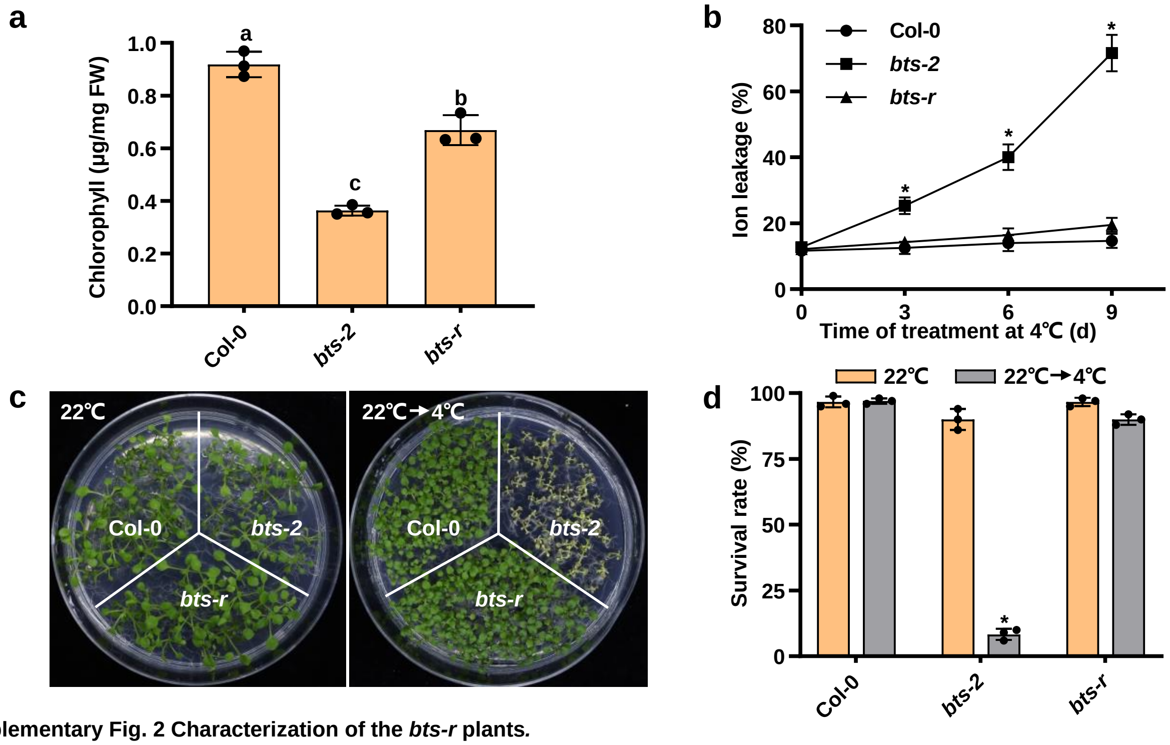

Supplementary Fig. 2 Characterization of the *bts-r* plants.

(**a**) Chlorophyll concentration of *bts-r* seedlings. Col-0, *bts-2,* and *bts-r* plants were grown at 22℃ for 4 weeks. Six plants for per replicate were collected and used for chlorophyll concentration measurement. Data presented as means ± SD (n=3 biological replicates) by one-way ANOVA with Tukey's HSD test (*P* ≤0.05).

(**b**) Ion leakage assays for *bts-r* plants. Plants were grown at 22℃ for 2 weeks, and then the plants were treated with 4℃ for the indicated times. Ten individual plant leaves were used for per replicate at each time point. Data presented as means ± SD (n=3 biological replicates) by two-factor ANOVA with Tukey's HSD test (**P* ≤0.05).

(**c**) and (**d**) Survival rate of Col-0, *bts-2,* and *bts-r* plants. Plants were grown at 22℃ for 2 weeks. Survival rates of these plants were measured at 7 days recovery after the treatment of 4℃ for additional 2 weeks (right panel). The plants grown at 22℃ for 4 weeks served as a control (left panel). Data presented as means ± SD (n=3 biological replicates) by two-factor ANOVA with Tukey's HSD test (**P* ≤0.05).

Source data are provided as a Source Data file.

Supplementary Fig.3

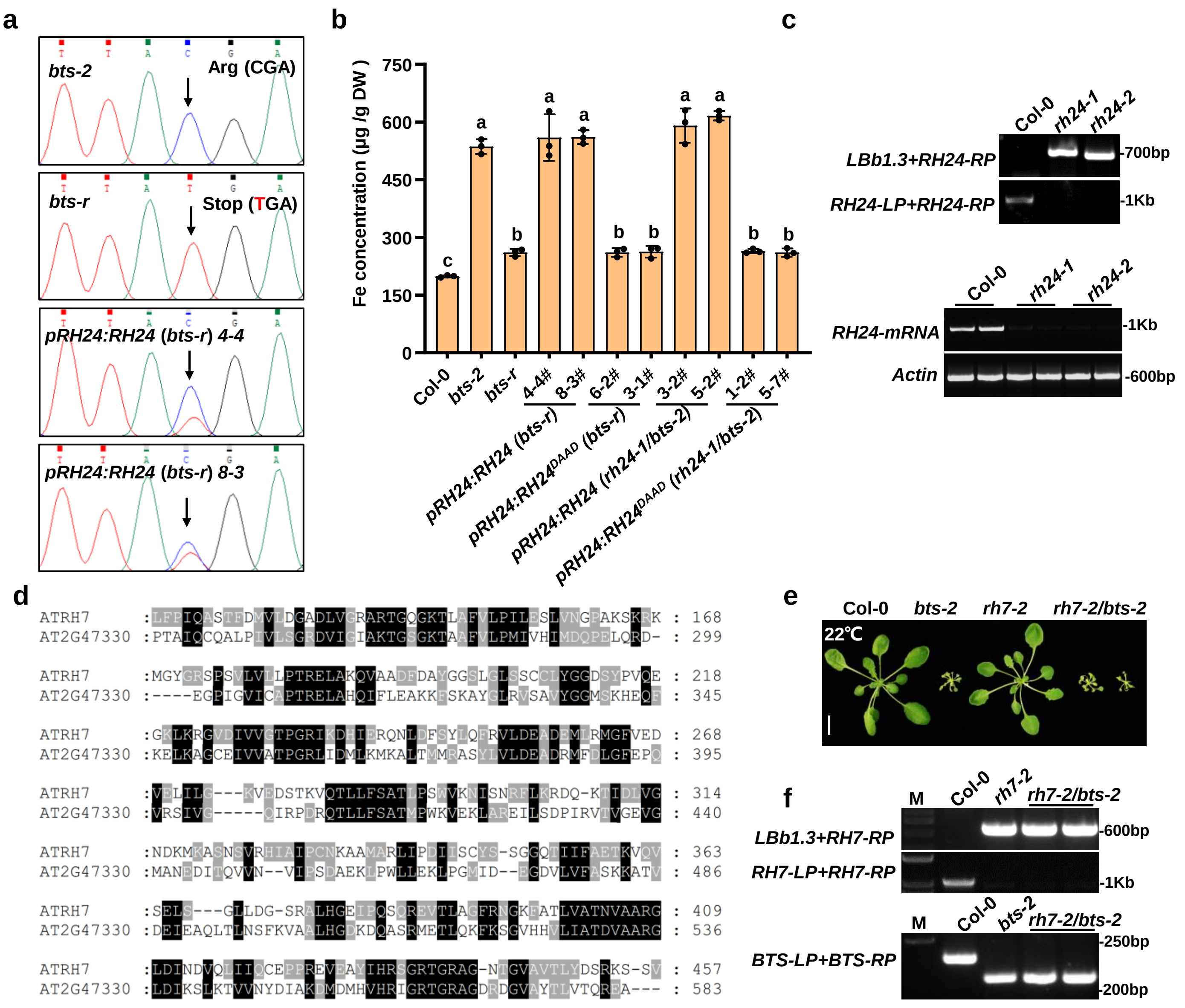

Supplementary Fig. 3 RH24 is required for iron accumulation in *bts-2* mutants.
(**a**) Sequencing analysis of *RH24* in *bts-2*, *bts-r*, and two complemented lines (4-4 and 8-3) of *bts-r* with *RH24.* The mutants were complemented with *RH24* driven by the native promoter.
(**b**) Iron concentration in Col-0, *bts-2, bts-r*, *pRH24*:*RH24* (*bts-r*), *pRH24*:*RH24*^{DAAD} (*bts-r*), *pRH24*:*RH24* (*rh24-1/bts-2*), and *pRH24*:*RH24*^{DAAD} (*rh24-1/bts-2*) plants. The plants were grown at 22℃ for 4 weeks, and then the leaves were sampled for Fe measurement. About twenty plants were used for each replicate. Data presented as means ± SD (n=3 biological replicates) by one-way ANOVA with Tukey's HSD test ($P \leq 0.05$). Source data are provided as a Source Data file.
(**c**) Identification of T-DNA insertion mutants *rh24-1* and *rh24-2*. The primers are in the primer list (Supplementary Data 2).
(**d**) The amino acid sequence alignment of RH24 (*AT2G47330*) and RH7. The alignment was performed using CLUSTAL W. The identical sequences are marked in black.
(**e**) Phenotype of *rh7-2/bts-2* double mutants. Col-0, *bts-2*, *rh7-2*, and *rh7-2/bts-2* plants were grown at 22℃ for 4 weeks. Bar =1 cm.
(**f**) Identification of T-DNA insertion for *rh7-2* and *rh7-2/bts-2* double mutants. The primers are in the primer list (Supplementary Data 2).

Supplementary Fig.4

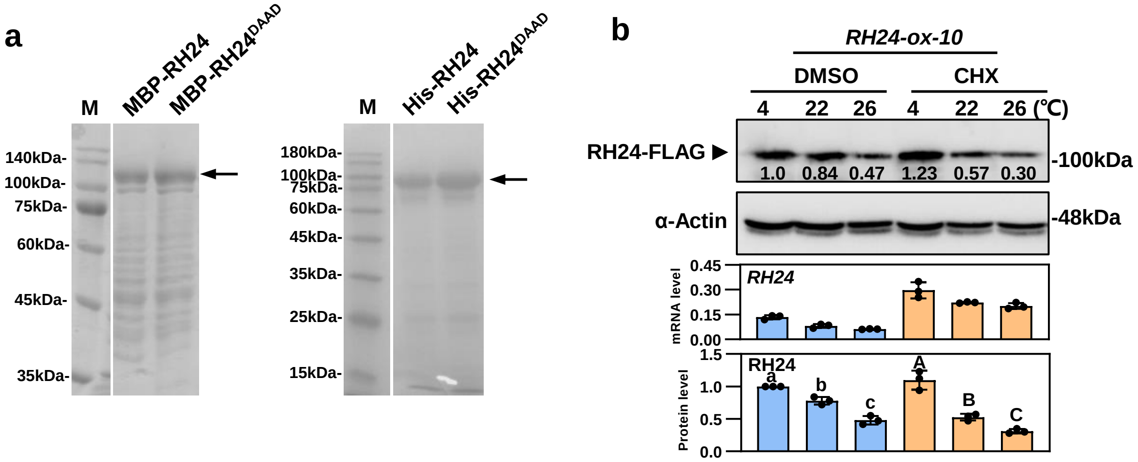

Supplementary Fig. 4 RH24 protein was accumulated under chilling stress.

(**a**) Recombinant MBP-RH24, MBP-RH24^{DAAD}, His-RH24, and His-RH24^{DAAD} proteins were shown by coomassie blue staining. M, protein ladder. Arrows indicate the proteins.

(**b**) Accumulation of RH24 protein was induced by chilling stress in 35S:*RH24-ox* plants. Plants were grown on ½ MS at 22℃ for 12 days, and then treated with 2 μM CHX at 4, 22, and 26℃ for 2 days, respectively. The protein levels of RH24 were examined by immunoblotting analysis and the expression levels of *RH24* were examined by RT-qPCR. The experiment was repeated three times with similar results. The band abundance was quantified by Image J. The relative protein level of RH24 was quantitatively analyzed. Different letters indicate the significant differences based on a two-factor ANOVA with Tukey's HSD test ($P \leq 0.05$, Data are means ± SD, n=3 biological repeats).

Source data are provided as a Source Data file.

Supplementary Fig.5

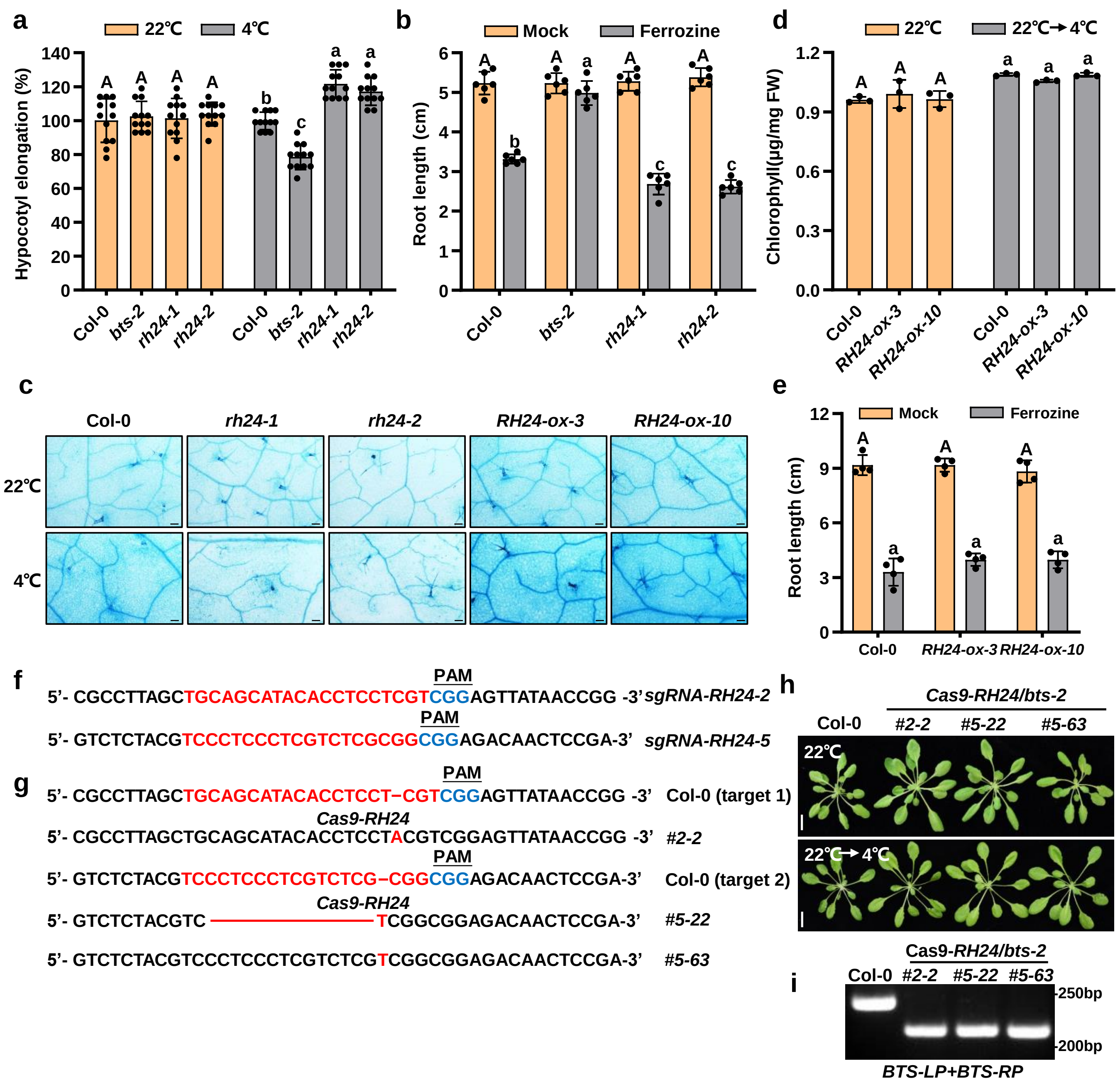

Supplementary Fig. 5 Phenotype of *rh24* mutant under cold and iron deficient conditions.
(**a**) Quantification of hypocotyl elongation in *rh24* mutants. Plants were gown in dark at 22℃ for 10 days or at 4℃ for 21 days. Hypocotyl elongation of the plants were compared with Col-0 at 22℃ or 4℃. The value of Col-0 was set as 100%. Data presented as means ± SD (n=12 biological replicates) by two-factor ANOVA with Tukey's HSD test ($P \leq 0.05$).
(**b**) Statistical analysis of root length for *rh24* mutants under iron deficiency. The plants were grown on ½ MS for 5 days and then transferred to ½ MS medium containing 100 μM Fe(II)-EDTA (mock) or 300 μM Ferrozine (iron deficiency) for 5 days at 22℃. The root length was measured. Data presented as means ± SD (n=6 biological replicates) by two-factor ANOVA with Tukey's HSD test ($P \leq 0.05$).
(**c**) Perls Fe staining for the leaves of Col-0, *rh24-1*, *rh24-2* and *RH24-ox* plants. The plants were grown in soil at 22℃ for 4 weeks (upper panel) and then transferred to 4℃ for another 7 days (lower panel). Bar = 200 μm. Images are representative of three independent experiments.
(**d**) Chlorophyll concentration of Col-0 and *RH24-ox* seedlings. The plants were grown at 22℃ for one week and then treated at 22℃ or 4℃ for another two weeks for chlorophyll measurement. Six plants were used for per replicate. Data presented as means ± SD (n=3 biological replicates) by two-factor ANOVA with Tukey's HSD test ($P \leq 0.05$).
(**e**) Statistical analysis of root length for *RH24-ox* plants under iron deficiency. The plants were grown on ½ MS for 5 days and then transferred to ½ MS medium containing 100 μM Fe(II)-EDTA (mock) or 300 μM Ferrozine (iron deficiency) for 7 days at 22℃. The root length was measured. Data presented as means ± SD (n=4 biological replicates) by two-factor ANOVA with Tukey's HSD test ($P \leq 0.05$).
(**f**) Schematic illustration of the two sgRNA:Cas9 targets and corresponding PAMs of *RH24*.
(**g**) The targeted sequences of the knockout mutants in three independent lines (#2-2, #5-22, and #5-63).
(**h**) The phenotype of the *Cas9-RH24/bts-2* double mutants. *RH24* was knocked out in *bts-2* plants by CRISPR-Cas9 approach. Three independent lines (#2-2, #5-22, and #5-63) were used. Col-0 and *Cas9-RH24/bts-2* plants were grown at 22℃ for 4 weeks (upper panel) or grown at 22℃ for 4 weeks then transferred to 4℃ for 7 days (lower panel). Bar =1 cm.
(**i**) Confirmation of *Cas9-RH24/bts-2* double mutants by PCR. The primers are in the primer list (Supplementary Data 2).
Source data are provided as a Source Data file.

Supplementary Fig.6

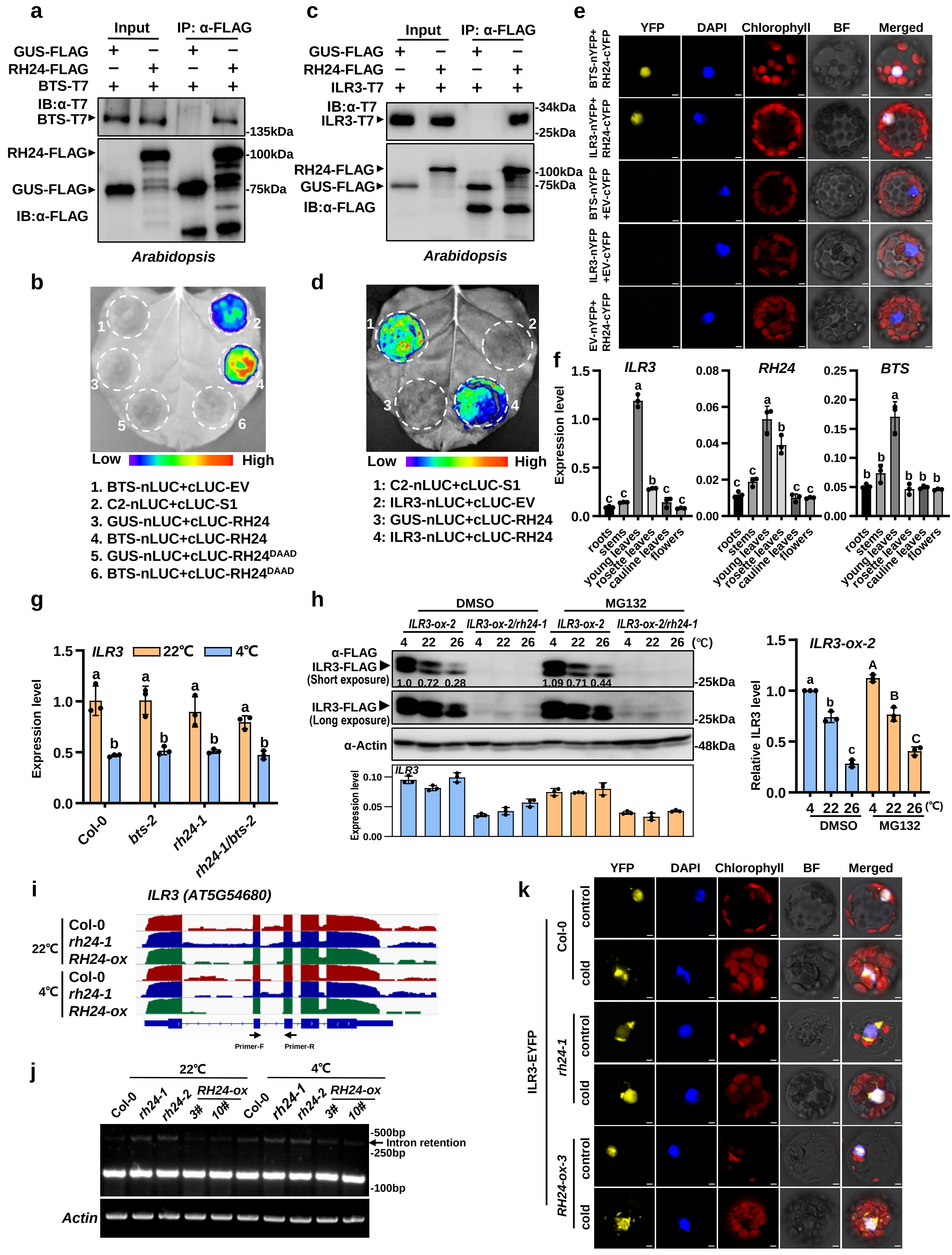

Supplementary Fig. 6 RH24 interacts with BTS and ILR3.
(**a**) RH24 interacts with BTS by co-immunoprecipitation (co-IP) assays. *35S:BTS-T7* and *35S:RH24-FLAG* were expressed in Arabidopsis protoplasts and were sampled for co-IP assays at 16 h after transfection.
(**b**) BTS interacts with RH24 *in vivo* by split-luciferase complementation (split-LUC) assays. *35S:BTS-nLUC* and *35S:cLUC-RH24* or *35S:cLUC-RH24*^{DAAD} were transiently expressed in *N. benthamiana* leaves. *35S:C2-nLUC* and *35S:cLUC-S1* were used as a positive control.
(**c**) RH24 interacts with ILR3 by co-IP assays. The protoplasts of Col-0 were used to express *35S:RH24-FLAG* and *35S:ILR3-T7*, and were subjected to co-IP assays at 16 h after transfection.
(**d**) RH24 interacts with ILR3 *in vivo* by split-LUC assays.
(**e**) RH24 interacts with BTS and ILR3 in BiFC assays. The proteins were expressed in Arabidopsis protoplast at 22℃ for 16 h. Nucleus were stained with DAPI. Chlorophyll was shown with autofluorescence. BF: bright field. Bar = 3 μm.
(**f**) *ILR3*, *RH24*, and *BTS* were highly expressed in young leaves. The expression levels of *RH24*, *ILR3*, and *BTS* in various tissues were examined by RT-qPCR. Data presented as means ± SD (n=3 biological replicates) by one-way ANOVA with Tukey's HSD test ($P \leq 0.05$).
(**g**) RH24 did not influence the transcription levels of *ILR3* in RT-qPCR assays. Two-week-old Col-0, *bts-2*, *rh24-1*, and *rh24-1/bts-2* seedlings were grown on ½ MS medium at 22℃, and then the plants were treated at 4℃ for 24 h. Data presented as means ± SD (n=3 biological replicates) by one-way ANOVA with Tukey's HSD test ($P \leq 0.05$).
(**h**) Accumulation of ILR3 is dependent on RH24. *ILR3-ox-2* and *ILR3-ox-2/rh24-1* lines were grown on ½ MS at 22℃ for 12 days and then were treated with 2.5 μM MG132 at 4, 22, or 26℃ for 2 days, respectively. The expression levels of *ILR3* were examined by RT-qPCR. The relative protein levels of ILR3 were quantitatively analyzed for three repeats (right panel). Data presented as means ± SD (n=3 biological replicates) by two-factor ANOVA with Tukey's HSD test ($P \leq 0.05$).
(**i**) The ILR3 pre-mRNA splicing with intron retention in Col-0, *rh24-1*, and *RH24-ox* plants. The intron retention of ILR3 pre-mRNA is visualized by the integrated genome (IGV) browser. The exon-intron structure of ILR3 is shown at the bottom. The positions of primers used for RT-PCR analysis are indicated by arrows.
(**j**) Mis-spliced transcript of ILR3 in Col-0, *rh24*, and *RH24-ox* plants. Plants were grown at 22℃ for 4 weeks and then transferred to 4℃ for 24 h. Arrows indicate the intron-retained transcripts.
(**k**) ILR3 was located in the nuclear bodies in the presence of RH24 under chilling stress. ILR3-EYFP were expressed in the protoplast of Col-0, *rh24-1*, and RH24-ox transgenic plants at 22℃ for 12 h and then were transferred to 22℃ (control) or 8℃ (cold) for another 1 h, respectively. Bar = 3 μm.
The above experiments were repeated three times with similar results. Source data are provided as a Source Data file.

Supplementary Fig.7

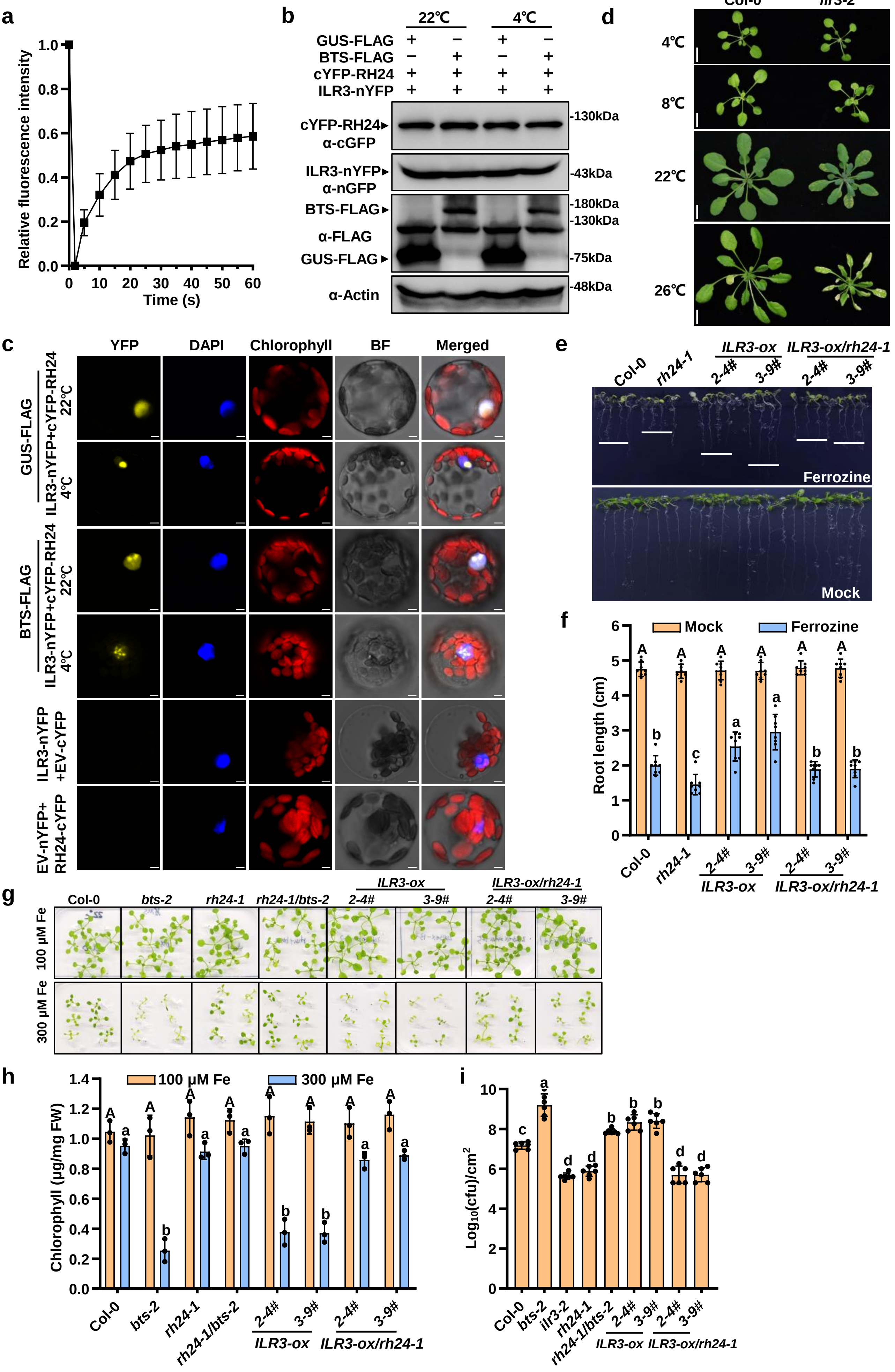

Supplementary Fig. 7 The chilling sensitivity and iron deficiency tolerance of *ILR3-ox* plants largely depend on RH24.

(**a**) FRAP recovery curves of ILR3-nYFP and cYFP-RH24. The proteins were transiently co-expressed in *N. benthamiana* leaves, and were suffered to photo bleach 48 h later . Data are shown as mean ± SD of 10 nuclear bodies.

(**b**) Cold treatment and BTS did not affect the protein levels of ILR3-nYFP and cYFP-RH24. The proteins were transiently expressed in *N. benthamiana* leaves at 22℃. Two days later, the plants were transferred to 4℃ for another 1h.

(**c**) BTS and cold treatments facilitate the condensate formation of ILR3-RH24 in Arabidopsis protoplast. The proteins were expressed in Arabidopsis protoplast at 22℃ for 12 h and then transferred to 22℃ or 4℃ for another 1 h. Bar = 3 μm.

(**d**) *ilr3-2* mutant is tolerant to chilling stress but not to higher temperature. Phenotypes of Col-0 and *ilr3-2* plants grown in soil at 22℃ for 2 weeks and then transferred to 4, 8, 22, or 26℃ for another 2 weeks, respectively. Bar =1 cm.

(**e**) Iron deficiency tolerance of *ILR3-ox* plants depended on RH24. Col-0, *rh24-1*, *ILR3-ox,* and *ILR3-ox/rh24-1* plants were grown on ½ MS for 5 d at 22℃ and then transferred to ½ MS medium containing 100 μM Fe(II)-EDTA (mock) or 300 μM Ferrozine (iron deficiency) for 5 days at 22℃.

(**f**) Statistical analysis of root length of the plants in (**e**). The root length of 10-day-old plants was measured. Data presented as means ± SD (n=8 biological replicates) by two-factor ANOVA with Tukey's HSD test ($P \leq 0.05$).

(**g**) Phenotypes of *ILR3-ox* and *ILR3-ox/rh24* plants under iron oversupply. Col-0, *bts-2*, *rh24-1*, *rh24-1/bts-2*, *ILR3-ox,* and *ILR3-ox/rh24-1* plants were grown on ½ MS medium containing 100 or 300 μM Fe(II)-EDTA for 14 days at 22℃, respectively.

(**h**) Chlorophyll concentration of the plants in (**g**). Six plants for per replicate were collected and used for chlorophyll concentration measurement. Data presented as means ± SD (n=3 biological replicates) by two-factor ANOVA with Tukey's HSD test ($P \leq 0.05$).

(**i**) Bacterial growth in Col-0, *bts-2*, *ilr3-2*, *rh24-1*, *rh24-1/bts-2*, *ILR3-ox,* and *ILR3-ox/rh24-1* plants. The leaves of 4-week-old plants were syringe infiltrated with *Pst* DC3000 at a concentration of 5×10^4 cfu/mL. The bacteria titer was determined at 4 days post inoculation. Six plants were used for per replicate. Data presented as means ± SD (n=6 biological replicates) by one-way ANOVA with Tukey's HSD test ($P \leq 0.05$).

Source data are provided as a Source Data file.
