# Supplementary material for: An RNA helicase coordinates with iron signal regulators to alleviate chilling stress in Arabidopsis
Source: Nat Commun. 2025 Apr 29;16:3988. doi: 10.1038/s41467-025-59334-9 (PMC12037725; doi:10.1038/s41467-025-59334-9)
Supplement: Supplementary file 2 — Description of Additional Supplementary Files [file 41467_2025_59334_MOESM2_ESM.pdf]

Description of Additional Supplementary Files

File Name: Supplementary Data 1.

Description: The CBF-regulated genes were not affected in *bts-2* mutants under chilling stress.

File Name: Supplementary Data 2.

Description: Primers used in the experiments.

File Name: Supplementary Data 3.

Description: The differential expression genes (DEGs) of iron metabolism pathways in the plants under 4℃.
